# Cross-sectional study of the ossification center of the C1–S5 vertebral bodies

**DOI:** 10.1007/s00276-012-1045-5

**Published:** 2012-11-29

**Authors:** Michał Szpinda, Mariusz Baumgart, Anna Szpinda, Alina Woźniak, Bogdan Małkowski, Marcin Wiśniewski, Celestyna Mila-Kierzenkowska, Dariusz Króliczewski

**Affiliations:** 1 Department of Normal Anatomy, The Nicolaus Copernicus University in Toruń, The Ludwik Rydygier Collegium Medicum in Bydgoszcz, Karłowicza 24 Street, 85-092 Bydgoszcz, Poland; 2 Department of Medical Biology, The Nicolaus Copernicus University in Toruń, The Ludwik Rydygier Collegium Medicum in Bydgoszcz, Karłowicza 24 Street, 85-092 Bydgoszcz, Poland; 3 Department of Positron Emission Tomography and Molecular Imaging, The Nicolaus Copernicus University in Toruń, The Ludwik Rydygier Collegium Medicum in Bydgoszcz, Karłowicza 24 Street, 85-092 Bydgoszcz, Poland

**Keywords:** Spine, Vertebral body ossification center, Dimensions, CT examination, Digital-image analysis, Skeletodysplasias, Human fetuses

## Abstract

**Purpose:**

Knowledge on the normative growth of the spine is relevant in the prenatal detection of its abnormalities. This study describes the size of the ossification center of C1–S5 vertebral bodies.

**Materials and methods:**

Using CT, digital-image analysis, and statistics, the size of the ossification center of C1–S5 vertebral bodies in 55 spontaneously aborted human fetuses aged 17–30 weeks was examined.

**Results:**

No sex significant differences were found. The body ossification centers were found within the entire presacral spine and in 85.5 % of S1, in 76.4 % of S2, in 67.3 % of S3, in 40.0 % of S4, and in 14.5 % of S5. All the values for the atlas were sharply smaller than for the axis. The mean transverse diameter of the body ossification center gradually increased from the axis to T12 vertebra, so as to stabilize through L1–L3 vertebrae, and finally was intensively decreasing to S5 vertebra. There was a gradual increase in sagittal diameter of the body ossification center from the axis to T5 vertebra and its stabilization for T6–T9 vertebrae. Afterward, an alternate progression was observed: a decrease in values for T10–T12 vertebrae, an increase in values for L1–L2 vertebrae, and finally a decrease in values for L3–S5 vertebrae. The values of cross-sectional area of ossification centers were gradually increasing from the axis to L2 vertebra and then started decreasing to S5 vertebra. The following cross-sectional areas were approximately equivalent to each other: for L5 and T3–T5, and for S4 and C1. The volumetric growth of the body ossification center gradually increased from the axis to L3 vertebra and then sharply decreased from L4 to S5.

**Conclusions:**

No male–female differences are found in the size of the body ossification centers of the spine. The growth dynamics for morphometric parameters of the body ossification centers of the spine follow similarly with gestational age.

## Introduction

The advancement of ultrasound, CT, MRI, and PET has revolutionized body imaging [8, 10, 17, 19]. Due to routine obstetric ultrasonography most fetal structures in utero can be assessed and commented on both the normal and the abnormal [9, 22, 23, 29, 30]. With the advent of three-dimensional ultrasound, the fetal spine has reliably been evaluated after the 12th week of pregnancy [7, 22]. The ossification timing of the spine has long been studied in detail with histologic [1, 3, 15, 20], radiographic [2], and ultrasound [6, 7] methods. This knowledge is a prerequisite for prenatal detection and exclusion of many structural spinal abnormalities, including caudal regression syndrome, hemivertebrae [6, 11, 14, 18, 27, 29, 30], butterfly vertebrae [21], diastematomyelia [27], and spina bifida [12, 16, 25]. Furthermore, delayed ossification centers are typical of osteochondrodysplasias [24, 26] and hypophosphatasia [31]. Except for the detailed morphometric study on the growing C4 vertebra in human fetuses, performed recently by Baumgart et al. [5], there has been no information about quantitative analysis of spinal ossification centers. In order to address this question specifically, in the present study we aimed

- to determine the size of the ossification center of C1–S5 vertebral bodies,
- to examine the influence of sex on the values obtained,
- to display graphically the relative growth of each parameter for the individual C1–S5 vertebrae.

## Materials and methods

**Fig. 1:**
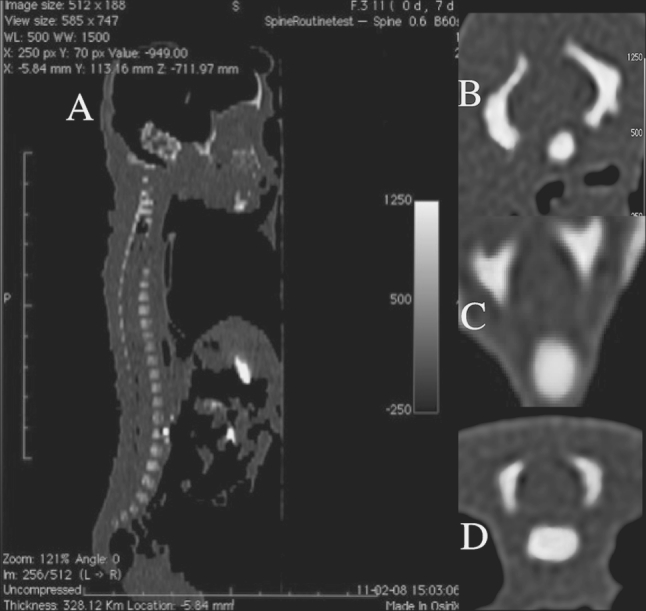
CT of a female fetus aged 24 weeks (in the sagittal projection) recorded in DICOM formats (*A*) with body ossification centers (in the transverse projection) of C4 (*B*), T6 (*C*), and L3 (*D*), being assessed by Osirix 3.9

This study encompassed 55 human fetuses (27 males and 28 females) aged 17–30 weeks of white racial origin (Table 1), which had been derived from spontaneous abortions or stillbirths in the years 1989–2001 as a result of placental insufficiency. Gestational age was determined by the crown–rump length [13]. No attempt was done to encourage fetal donation. The use of the fetuses for research was approved by the University Research Ethics Committee (KB 275/2011). The fetuses included were free from visible external malformations. The entire material was immersed in 10 % neutral buffered formalin solution with ethanol added. After having been fixed in formalin, the fetuses underwent CT examinations with the reconstructed slice width option of 0.4 mm. As a consequence, 128 slices were acquired simultaneously by biograph mCT (Siemens). No bones showed evidence of abnormal development. The scans obtained were recorded in DICOM formats (Fig. 1a), which enabled us to compute three-dimensional reconstructions and the morphometric analysis of chosen objects. The gray scale of obtained CT images in Hounsfield units ranged widely, attaining the following values: from −275 to −134 for a minimum and from +1,165 to +1,558 for a maximum. As a result, the window width (WW) varied from 1,404 to 1,692, whereas the window level (WL) reached the values from +463 to +712. Such a wide WW, being characterized for osseous structures, enabled us both to estimate precisely the borders of each body ossification center of the spine and to determine accurate values for the parameters studied. DICOM formats were assessed by digital-image analysis of Osirix 3.9 (Fig. 1b), which semi-automatically calculated linear (sagittal and transverse diameters), two-dimensional (cross-sectional area), and three-dimensional (volume) parameters of the ossification center of C1–S5 vertebral bodies (Fig. 1c, d). The contouring procedure for each body ossification center was outlined with a cursor and stored.

**Table 1:** Distribution of the fetuses studied

Gestational age (weeks)	Crown– rump length (mm)				Number	Sex	
	Mean	SD	Min	Max		Male	Female
17	115.00		115.00	115.00	1	0	1
18	133.33	5.77	130.00	140.00	3	1	2
19	149.50	3.82	143.00	154.00	8	3	5
20	161.00	2.71	159.00	165.00	4	2	2
21	174.75	2.87	171.00	178.00	4	3	1
22	185.00	1.41	183.00	186.00	4	1	3
23	197.60	2.61	195.00	202.00	5	2	3
24	208.67	3.81	204.00	213.00	9	5	4
25	214.00		214.00	214.00	1	0	1
26	229.00	5.66	225.00	233.00	2	1	1
27	239.17	3.75	235.00	241.00	6	6	0
28	249.50	0.71	249.00	250.00	2	0	2
29	253.00	0.00	253.00	253.00	2	0	2
30	263.25	1.26	262.00	265.00	4	3	1
Total					55	27	28

**Table 2:** Morphometric parameters of the ossification center of vertebral bodies C1–S5

Vertebra	Vertebral body ossification center							
	Transverse diameter (mm)		Sagittal diameter (mm)		Cross-sectional area (mm^2^)		Volume (mm^3^)	
	Mean	SD	Mean	SD	Mean	SD	Mean	SD
C1	0.91	1.52	0.52	0.86	1.47	2.69	1.80	3.30
C2	2.75	0.79	1.97	0.50	4.91	2.23	6.89	3.02
C3	2.80	0.69	2.19	0.51	5.23	2.26	7.14	3.11
C4	2.77	0.68	2.38	0.56	5.50	2.36	7.55	2.62
C5	3.00	0.68	2.54	0.52	6.26	2.52	8.50	3.47
C6	3.22	0.90	2.75	0.47	7.27	3.24	9.77	3.92
C7	3.45	0.82	2.95	0.54	7.88	3.01	10.80	4.00
T1	3.84	0.90	3.13	0.52	9.33	3.46	12.40	4.80
T2	4.11	1.07	3.42	0.58	11.13	4.63	14.61	6.23
T3	4.31	0.90	3.58	0.60	11.59	4.27	15.63	6.32
T4	4.30	0.86	3.65	0.61	11.89	4.38	15.65	5.92
T5	4.32	0.97	3.67	0.73	11.61	4.78	15.74	6.33
T6	4.41	1.05	3.63	0.87	12.21	4.54	16.44	6.15
T7	4.52	1.07	3.73	0.91	12.67	5.41	17.66	6.88
T8	4.59	1.06	3.73	0.86	12.91	5.46	17.32	6.55
T9	4.67	1.09	3.77	0.91	12.73	5.06	17.34	6.62
T10	4.78	1.05	3.57	0.69	12.55	4.66	17.35	6.69
T11	4.86	1.18	3.51	0.61	13.56	5.74	18.56	7.69
T12	4.97	1.29	3.46	0.60	14.00	6.16	19.46	8.66
L1	4.94	1.60	3.61	0.61	14.69	6.38	20.77	8.88
L2	4.89	1.63	3.86	0.83	15.98	7.95	21.29	12.67
L3	4.82	1.71	3.77	0.96	15.48	7.96	21.50	10.95
L4	4.59	1.62	3.61	1.07	13.80	7.82	19.78	13.11
L5	4.19	1.45	3.47	1.03	11.98	7.11	16.93	11.04
S1	3.31	1.92	2.61	1.48	8.60	6.78	11.10	8.66
S2	2.82	1.89	2.05	1.34	5.72	5.21	7.56	6.91
S3	2.11	1.81	1.54	1.27	3.67	3.95	4.38	5.03
S4	1.14	1.64	0.81	1.17	1.69	2.89	1.91	3.54
S5	0.18	0.65	0.15	0.54	0.19	0.84	0.07	0.54

The four following features of the ossification center of every vertebral body were assessed for each fetus:

1. transverse diameter (in mm), corresponding to the distance between the left and right borderlines of the ossification center (in transverse projection),
2. sagittal diameter (in mm), corresponding to the distance between the anterior and posterior borderlines of the ossification center (in sagittal projection),
3. cross-sectional area (in mm^2^), traced around the ossification center (in transverse projection), and
4. volume (in mm^3^).

In a continuous effort to minimize measurements and observer bias, all the measurements were performed by one researcher (M.B). Each measurement was repeated three times under the same conditions, and the mean of the three was considered as definitive. The results obtained in the number of 6,380 were subjected to statistical analysis. The intra-observer variation was evaluated by the one-way ANOVA test for paired data.

The data obtained were checked for normality of distribution using the Kolmogorov–Smirnov test and for homogeneity of variance with the use of Levene’s test. For statistics, the fetuses were separated as follows: group I (17–19 weeks) 12 specimens, group II (20–23 weeks) 17 specimens, group III (24–27 weeks) 18 specimens, and group IV (28–30 weeks) 8 specimens. As a consequence of the statistical analysis, Student’s *t* test was used to examine whether or not sex influenced the values obtained. To examine sex differences, we checked possible differences between the four following age groups: 17–19, 20–23, 24–27, and 28–30 weeks. In turn, we tested sex differences for the entire group, without taking into account fetal ages. By plotting the numerical data of each parameter of the ossification center versus the corresponding vertebra, we obtained curves for their relative growth.

## Results

In the examined material, all the ossification centers of the presacral spine were visualized. This stood out in stark contrast when compared to the sacral ossification centers, being visible in 47 (85.5 %) fetuses for S1, 42 (76.4 %) fetuses for S2, 37 (67.3 %) fetuses for S3, 22 (40.0 %) fetuses for S4, and 8 (14.5 %) fetuses for S5.

**Fig. 2:**
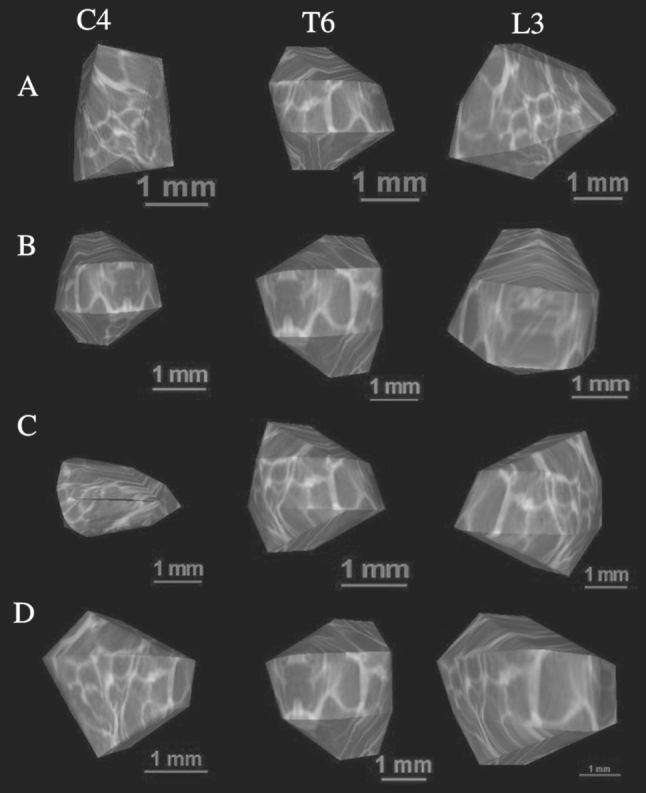
Ossification centers of the vertebral bodies C4, T6, and L3 in fetuses aged 18 weeks (*A*), 21 weeks (*B*), 25 weeks (*C*), and 29 weeks (*D*)

The morphometric values obtained were characterized by normality of distribution (the Kolmogorov–Smirnov test) and homogeneity of variance (Levene’s test). No statistically significant differences were found in evaluating intra-observer reproducibility of the spinal measurements (*P* > 0.05, the one-way ANOVA test for paired data and post hoc RIR Tukey test). In addition, since no significant difference was observed in the values of the parameters studied according to sex (*P* > 0.05, Student’s *t* test), no attempt was made to further separate the results obtained according to sex (Table 2). By contrast, advancing gestational age was characterized by a statistically significant increase (*P* = 0.0000, the one-way ANOVA test for unpaired data and post hoc RIR Tukey test) in values of all measurements. Figure 2 presents the body ossification centers for C4, T6, and L3 in fetuses aged 18 (A), 21 (B), 25 (C), and 29 weeks (D).

**Fig. 3:**
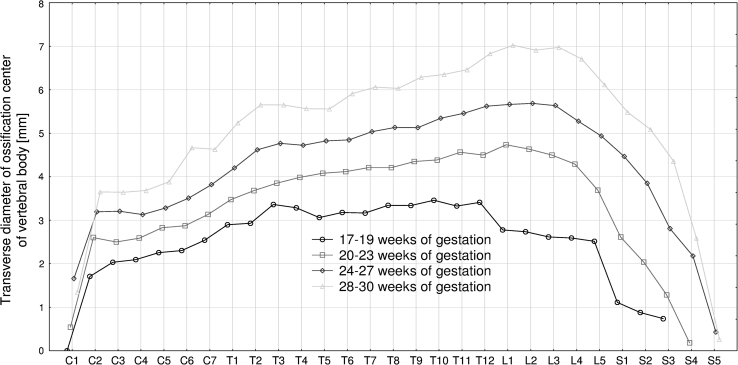
Mean transverse diameters of body ossification centers of the individual vertebrae in fetuses aged: 17–19, 20–23, 24–27, and 28–30 weeks of gestation

**Fig. 4:**
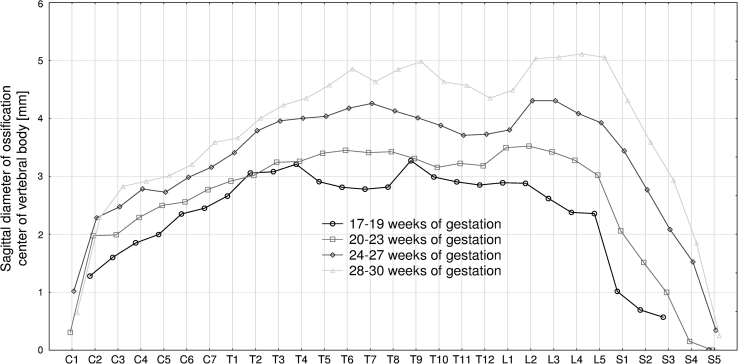
Mean sagittal diameters of body ossification centers of the individual vertebrae in fetuses aged: 17–19, 20–23, 24–27, and 28–30 weeks of gestation

**Fig. 5:**
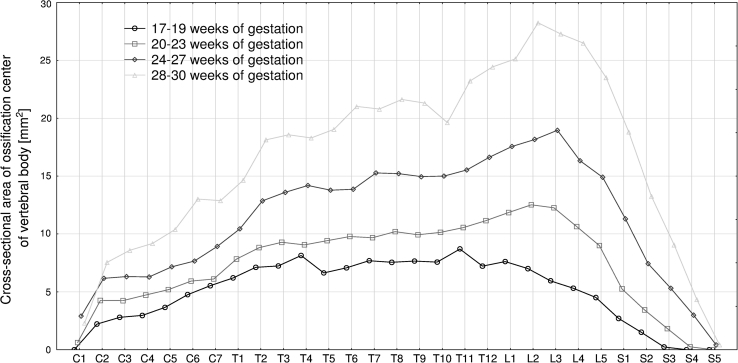
Mean cross-sectional areas of body ossification centers of the individual vertebrae in fetuses aged: 17–19, 20–23, 24–27, and 28–30 weeks of gestation

**Fig. 6:**
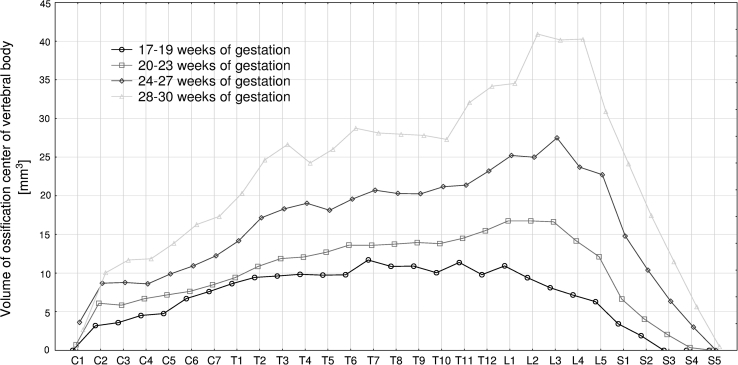
Mean volumes of body ossification centers of the individual vertebrae in fetuses aged: 17–19, 20–23, 24–27, and 28–30 weeks of gestation

The four following figures display the patterns for growth in transverse diameter (Fig. 3), sagittal diameter (Fig. 4), cross-sectional area (Fig. 5), and volume (Fig. 6) of the ossification center of the individual C1–S5 vertebrae in fetuses aged 17–19, 20–23, 24–27, and 28–30 weeks. The growth dynamics for each parameter studied followed similarly in the four age groups. In all, the values for the atlas were sharply smaller than for the axis, being expressed by the following means: 0.91 ± 1.52  versus 2.75 ± 0.79 mm for transverse diameter, 0.52 ± 0.86  versus 1.97 ± 0.50 mm for sagittal diameter, 1.47 ± 2.69  versus 4.91 ± 2.23 mm^2^ for cross-sectional area, and 1.80 ± 3.30 versus 6.89 ± 3.02 mm^3^ for volume.

The mean transverse diameter of the body ossification center gradually increased from the axis (2.75 ± 0.79 mm) to T12 vertebra (4.97 ± 1.29 mm), so as to stabilize through L1−L3 vertebrae (4.94 ±1.60 mm, 4.89 ± 1.63 mm, 4.82 ± 1.71 mm). Then, it was intensively decreasing to reach the value of 0.18 ± 0.65 mm for S5 vertebra. The value for vertebra S1 (3.31 ± 1.92 mm) was approximately equivalent to that of C6 vertebra (3.22 ± 0.90 mm).

There was a gradual increase in sagittal diameter of the body ossification center from the axis (1.97 ± 0.50 mm) to T5 vertebra (3.67 ± 0.73 mm), and its stabilization for T6–T9 vertebrae (3.63 ± 0.87 mm, 3.73 ± 0.91 mm, 3.73 ± 0.86 mm, 3.77 ± 0.91 mm). Afterward, an alternate progression was observed as follows: a decrease in values for T10–T12 vertebrae (3.57 ± 0.69 mm, 3.51 ± 0.61 mm. 3.46 ± 0.60 mm), an increase in values for L1 (3.61 ± 0.61 mm) and L2 (3.86 ± 0.83 mm) vertebrae, and finally a decrease in values for L3 (3.77 ± 0.96 mm) — S5 (0.15 ± 0.54 mm) vertebrae.

The growth dynamics for the cross-sectional area paralleled that of the volume of the body ossification center. The values of its cross-sectional area gradually increased from the axis (4.91 ± 2.23 mm^2^) to L2 vertebra (15.98 ± 7.95 mm^2^) and then started decreasing to reach the value of 0.19 ± 0.84 mm^2^ for S5 vertebra. The following cross-sectional areas were approximately equivalent to each other: for L5 (11.98 ± 7.11 mm^2^) and T3–T5 (11.59 ± 4.27, 11.89 ± 4.38, 11.61 ± 4.78 mm^2^), and for S4 (1.69 ± 2.89 mm^2^) and C1 (1.47 ± 2.69 mm^2^).

The volumetric growth of the body ossification center gradually increased from the axis (6.89 ± 3.02 mm^3^) to L3 vertebra (21.50 ± 10.95 mm^3^). Next, there was a sharp decrease in its volume from L4 (19.78 ± 13.11 mm^3^), through S1 (11.10 ± 8.66 mm^3^) and S3 (4.38 ±5.03 mm^3^) to S5 (0.07 ± 0.54 mm^3^). The following volumes of the body ossification centers were approximately equivalent to each other: for C1 (1.80 ± 3.30 mm^3^) and S4 (1.91 ± 3.54 mm^3^), for C4 (7.55 ± 2.62 mm^3^) and S2 (7.56 ± 6.91 mm^3^), for C7 (10.80 ± 4.00 mm^3^) and S1 (11.10 ± 8.66 mm^3^), and for T8–T10 (17.32 ± 6.55, 17.34 ± 6.62, 17.35 ± 6.69 mm^3^) and L5 (16.93 ± 11.04 mm^3^).

## Discussion

The present study attempts to extend the existing literature relating to development of the spine in human fetuses. The evidence material consisted of four results for each vertebra, thereby 116 results for each fetus, resulting in 6,380 numerical data for the entire series. The values for the ossification centers of the vertebral bodies in the material under examination could be considered as both normative and real, because of the following six reasons. Firstly, the fetuses comprised a numerous (*n* = 55) sample size without any visible non-osseous or osseous malformations. Secondly, tissue shrinkage related to formalin immersion had no influence on the values obtained [4, 5]. Thirdly, valid objectives methods (Biograph mCT, Osirix 3.9) were used for assessing all the parameters, with the greatest accuracy in measuring the selected dimensions to the nearest 0.01 mm. Fourthly, the four parameters studied were precise and clearly definable. Fifthly, the wide window width (1,404–1,692) of obtained CT images enabled us both to estimate precisely the borders of each body ossification center of the spine and to determine accurate values for the parameters studied. Finally, in our material, all the calculations were based on direct measurements, instead of deduced, extrapolated through a series of indirect measurements.

Although some authors [28] had reported significant sex differences, resulting in a slightly more rapid rate of ossification in female than in male fetuses, our previous [4, 5] and present results did not support that finding. On the contrary, the numerical data obtained in our series were not under the influence of sex that made us present them without regard to sex.

It is apparent that growth of the ossification centers is three-dimensional with simultaneous growth in transverse and sagittal diameters, cross-sectional area, and volume with advancing fetal age. According to Baumgart et al. [5], in fetuses aged 17–30 weeks of gestation, the ossification center of the C4 vertebral body grew logarithmically in both transverse (*y* = –8.836 + 3.708 × ln(Age) ± 0.334, *R* ^2^ = 0.76) and sagittal (*y* = –7.748 + 3.240 × ln(Age) ± 0.237; *R* ^2^ = 0.83) diameters and linearly in both cross-sectional area (*y* = –4.690 + 0.437 × Age ±1.172; *R* ^2^ = 0.63) and volume (*y* = –5.917 + 0.582 × Age ± 1.157; *R* ^2^ = 0.77). In our opinion, even a better understanding of spinal growth patterns may be gained by studying all the individual C1–S5 vertebrae in every specimen at the same time, as has been provided in this study.

On the whole, the shape of the curves representing the values for the four examined parameters was similar in any age range. Firstly, there was a sharp increase in values between the atlas and the axis. The ossification center of the C1 vertebral body was considerably smaller than that of the axis. To our knowledge, this result is not surprising, because the C1 vertebral body fuses onto that of C2 to become its dens, with no weight bearing function. Secondly, there was a gradual increase in all the values from the axis until T5 vertebra for the sagittal diameter, T12 vertebra for the transverse diameter, L2 vertebra for the cross-sectional area, and finally L3 vertebra for the volume of the body ossification center. This suggests that the body ossification center grew much faster in sagittal diameter than transverse one, thereby contributing to its shape. Furthermore, the volumetric growth reached a maximum value for L3, while the cross-sectional area for L2 of the ossification center. The largest values of all the examined parameters were related to the lower thoracic–upper lumbar vertebrae. In our opinion, this may be a direct consequence of the timing of ossification, since the vertebral bodies ossify in a predictable pattern, starting with the inferior thoracic–superior lumbar part of the spine [3, 20]. From there, the ossification sequence progresses in both cranial and caudal directions. Such a considerable increase in the body ossification centers within the inferior thoracic–superior lumbar segment, observed in the material under examination, closely corresponded with the parallel increase in size of the vertebral bodies reported by Schild et al. [22]. This may be in part associated with the postnatal need to withstand greater stresses and strains. Thirdly, a phase of stabilized values occurred at the levels of L1–L3 for the transverse diameter and T6–T9 for the sagittal diameter. Fourthly, there was a sharp decrease in all the values of the sacral segment. According to the professional literature [6], this may result from the delayed appearance of sacral ossification centers. As reported by Biasio et al. [6], in fetuses aged 17 weeks, the body ossification centers of the sacrum were present in all fetuses for S1 and S2, in 75 % for S3, in 12.5 % for S4, and in no one fetus for S5. In the material under examination, the sacral vertebrae were visualized and assessed as follows: S1, in 47 (85.5 %) fetuses; S2, in 42 (76.4 %) fetuses; S3, in 37 (67.3 %) fetuses; S4, in 22 (40.0 %) fetuses; and finally S5, only in 8 (14.5 %) fetuses.

The ability to recognize both spinal and neural tube defects in fetuses using ultrasonography is based on an understanding of the normal appearance of the three primary ossification centers within each vertebra: one in the body and two in the neural arch [7]. It should be emphasized, however, that the sacral bodies normally ossify before the sacral arches. On the contrary, delayed sacral body ossification with respect to the sacral arches is typical of achondrogenesis [24, 28]. Caudal regression syndrome is a spinal abnormality ranging from isolated sacral agenesis to the absence of the lumbosacral spine [6]. Hemivertebra lacks one of the two chondrification centers which normally fuse onto one ossification center. As a result, the defective vertebra acts as a triangular wedge-shaped ossified structure, causing contralateral spine deviation [27]. Butterfly vertebra results from the failure of fusion of two chondrification centers, being separated by the persistent notochord [21]. Diastematomyelia as a spinal dysraphism is characterized by a sagittal cleft of the spinal cord with splaying of vertebral arches [18, 27]. Spina bifida occurs when the neural arches of the lumbosacral spine failed to fuse allowing the spinal cord to protrude through an opening [12, 16]. A delayed appearance of ossification centers and widespread demineralization is typical of osteogenesis imperfecta type II [26], achondrogenesis [24], and hypophosphatasia [31].

The present study is the first to provide objective information on the quantitative growth of body ossification centers of the entire spine. The main limitation of the present study has resulted from a relatively narrow fetal age, varying from 17 to 30 weeks of gestation. Besides, all measurements have been done by one observer in a blind fashion. Furthermore, our findings have been presented as if describing a developmental sequence in one fetus, even though the numerical data have truly been cross-sectional, derived from 55 autopsied fetuses.

## Conclusions

1. No male–female differences are found in the size of the body ossification centers of the spine.
2. The growth dynamics for morphometric parameters of the body ossification centers of the spine follow similarly with gestational age.
